# Antibacterial Bisabolane-Type Sesquiterpenoids from the Sponge-Derived Fungus *Aspergillus* sp.

**DOI:** 10.3390/md10010234

**Published:** 2012-01-19

**Authors:** Dan Li, Ying Xu, Chang-Lun Shao, Rui-Yun Yang, Cai-Juan Zheng, Yi-Yan Chen, Xiu-Mei Fu, Pei-Yuan Qian, Zhi-Gang She, Nicole J. de Voogd, Chang-Yun Wang

**Affiliations:** 1 Key Laboratory of Marine Drugs, Ministry of Education, School of Medicine and Pharmacy, Ocean University of China, Qingdao 266003, China; Email: lidaneasy@163.com (D.L.); caijuan2002@163.com (C.-J.Z.); yiyanchen80@hotmail.com (Y.-Y.C.); fuxiumei2005@yahoo.com.cn (X.-M.F.); 2 KAUST Global Academic Partnership Program, Division of Life Science, The Hong Kong University of Science and Technology, Clear Water Bay, Hong Kong, China; Email: boxuying@ust.hk (Y.X.); boqianpy@ust.hk (P.-Y.Q.); 3 Key Laboratory for the Chemistry and Molecular Engineering of Medicinal Resources, Ministry of Education, School of Chemistry & Chemical Engineering, Guangxi Normal University, Guilin 541004, China; Email: yang_rui_yun@163.com; 4 School of Chemistry and Chemical Engineering, Sun Yat-sen University, Guangzhou 510275, China; Email: cesshzhg@mail.sysu.edu.cn; 5 Netherlands Centre for Biodiversity Naturalis, P.O. box 9517, Leiden 2300 RA, The Netherlands; Email: voogd@naturalis.nnm.nl

**Keywords:** bisabolane-type sesquiterpenoid, antibacterial, sponge-derived fungus, *Aspergillus* sp.

## Abstract

Four new bisabolane-type sesquiterpenoids, aspergiterpenoid A (**1**), (−)-sydonol (**2**), (−)-sydonic acid (**3**), and (−)-5-(hydroxymethyl)-2-(2′,6′,6′-trimethyltetrahydro-2H- pyran-2-yl)phenol (**4**) together with one known fungal metabolite (**5**) were isolated from the fermentation broth of a marine-derived fungus *Aspergillus* sp., which was isolated from the sponge *Xestospongia testudinaria* collected from the South China Sea. Four of them (**1**–**4**) are optically active compounds. Their structures and absolute configurations were elucidated by using NMR spectroscopic techniques and mass spectrometric analysis, and by comparing their optical rotations with those related known analogues. Compounds **1**–**5** showed selective antibacterial activity against eight bacterial strains with the MIC (minimum inhibiting concentrations) values between 1.25 and 20.0 µM. The cytotoxic, antifouling, and acetylcholinesterase inhibitory activities of these compounds were also examined.

## 1. Introduction

Several bisabolane-type phenolic sesquiterpenoids have been isolated from marine materials, such as the gorgonian coral *Pseudopterogorgia rigida* [1], the sponges *Didiscus flavus* [2] and *Myrmekioderma styx* [3], the marine-derived fungi *Verticillium tenerum* [4], *Aspergillus* sp. [5] and *Aspergillus sydowii* [6]. These compounds have received much attention because of their multiple potent biological activities including the acetylcholinesterase inhibitory activity [7], antioxidant activity [8], cytotoxic activity [9], and antibacterial activity [10].

As part of our ongoing investigation on new natural antibacterial, cytotoxic, and nontoxic antifouling agents from marine fungi in the South China Sea [5,11,12,13], the EtOAc extract of a fermentation broth of the fungus *Aspergillus* sp. isolated from the sponge *Xestospongia testudinaria* attracted our attention because the extract showed significant antibacterial activity. The crude EtOAc extract of the fungal culture was subjected to silica gel column chromatography, Sephadex LH-20 and further semi-preparative HPLC, and this led to the isolation of five bisabolane-type sesquiterpenoids, including four new compounds, aspergiterpenoid A (**1**), (−)-sydonol (**2**), (−)-sydonic acid (**3**), (−)5-(hydroxymethyl)-2-(2′,6′,6′-trimethyltetrahydro-2H-pyran-2-yl)phenol (**4**), and one known compound, (*Z*)-5-(hydroxymethyl)-2-(6′-methylhept-2′-en-2′-yl)phenol (**5**) [14]. Their structures were elucidated using NMR spectroscopic techniques and mass spectrometric analysis, and the absolute configurations of compounds **1**–**3** were confirmed as *R* by comparing the optical rotations with (+)-sydonic acid, while compound **4** was confirmed as *S* using the same method. The antibacterial, cytotoxic, antifouling, and acetylcholinesterase inhibitory activities of these compounds (**1**–**5**) were examined. Compounds **1**–**5** exhibited selective antibacterial activity against six pathogenic bacteria and two marine bacteria. Compound **4** showed a significant inhibitory effect on larval settlement of the barnacle *Balanus amphitrite*, while compound **5** was found to have an obvious toxic effect on the larvae.

## 2. Results and Discussion

**Figure 1:**
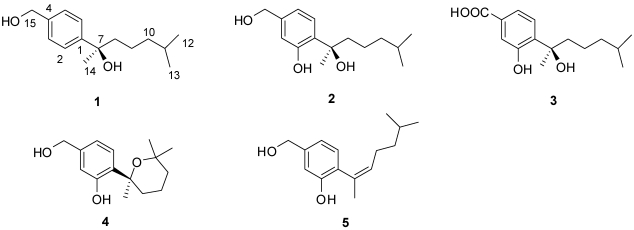
Structures of compounds **1–5**.

Aspergiterpenoid A (**1**) was isolated as optically active white powder, [α]^25^_D_ −4.7 (*c* 3.21, CHCl_3_). The molecular formula of C_15_H_24_O_2_ was confirmed by HREIMS that displayed an [M]^•+^ *m*/*z* of 236.1769 (calcd. 236.1771). The IR absorption bands indicated the existence of hydroxyl functionality (3397 cm^−1^). The ^1^H NMR spectrum of **1** showed two exchangeable proton signals at δ_H_ 1.66 and 1.71 (validated by D_2_O change assay), one A_2_B_2_ spin system assignable to a 1,4-disubstitued benzene ring at δ_H_ 7.43 (2H, dd, 8.4, 1.8), 7.34 (2H, dd, 8.4, 1.8) (Table 1). The ^1^H NMR spectrum revealed the presence of other signals including two doublet methyl groups [H_3_-12 (δ_H_ 0.81), H_3_-13 (δ_H_ 0.81), one singlet methyl group [H_3_-14 (δ_H_ 1.55)], four methylenes [H_2_-15 (δ_H_ 4.69), H_2_-8 (δ_H_ 1.77), H_2_-9 (δ_H_ 1.12 and 1.26), and H_2_-10 (δ_H_ 1.12)], and one methine [H-11 (δ_H_ 1.47)]. Thirteen carbon signals observed in the ^13^C NMR spectrum (Table 1) indicated that there is a 1,4-disubstitued benzene ring in compound **1**. Detailed analysis of the ^1^H and ^13^C NMR spectra revealed that **1** is a bisabolane-type sesquiterpenoid, which is similar to sydonol [15]. The main difference between the spectra of **1** and sydonol was the A_2_B_2_ spin system in **1** instead of ABX spin system in sydonol, suggesting that the phenolic hydroxyl group was missing in **1**. Detailed assignments for carbons and protons were unambiguously accomplished by analysis of 2D NMR spectral data, and the planar structure of **1** was confirmed and is shown in Figure 1. Interestingly, the optical rotation value for **1** was [α]^25^_D_ −4.7 (*c* 3.21, CHCl_3_), contrary to that of sydonol ([α]^20^_D_ +7.2 (*c* 1.0, MeOH)) [15]. On the basis of the absolute configurations of the co-metabolites **2** and **3** and a shared biogenesis with these two compounds, the configuration of **1** should be assigned as 7*R*. It is noteworthy to mention that metabolite **1** represents the rare example of a bisabolane-type sesquiterpenoid possessing a 1,4-disubstitued benzene ring isolated from marine organisms.

**Table 1:** NMR spectroscopic data (600 MHz, CDCl_3_) of Aspergiterpenoid A (**1**).

Position	δ_C_, mult	δ_H_ (*J* in Hz)
1	147.8, C	–
2	125.1, CH	7.43, dd (8.4, 1.8)
3	127.0, CH	7.34, dd (8.4, 1.8)
4	139.1, C	–
5	127.0, CH	7.34, dd (8.4, 1.8)
6	125.1, CH	7.43, dd (8.4, 1.8)
7	74.8, C	–
8	44.4, CH_2_	1.77, m
9	21.8, CH_2_	1.26, m; 1.12, overlapped
10	39.3, CH_2_	1.12, overlapped
11	27.9, CH	1.47, m
12	22.6, CH_3_	0.81, d (6.6)
13	22.7, CH_3_	0.81,d (6.6)
14	30.3, CH_3_	1.55, s
15	65.2, CH_2_	4.69, d (3.0)
7–OH		1.71, s
15–OH		1.66, brs

The spectroscopic data of compounds **2** and **3** were almost identical to those of (+)-sydonol and (+)-sydonic acid, respectively. Their opposite optical rotation values indicated that **2** and **3** were the enantiomers of (+)-sydonol and (+)-sydonic acid, respectively. (+)-Sydonol possessing the positive optical rotation value, ([α]^20^_D_ +7.2 (*c* 1.0, MeOH)), was isolated from a terrestrial *Aspergillus* sp. by Nukina and co-workers in 1981 and exhibited antifungal activity against *Cochliobolus lunata* (IFO6299) [15]. Sydonic acid was isolated from the fungus *A. sydowi* in 1978 as a racemate [16], while (+)-sydonic acid ([α]^25^_D_ +2.73 (*c* 2.30, MeOH)) was isolated from *Glonium* sp. in 2009 [17]. However, in our case, the optical rotation values of **2** and **3** were [α]^25^_D_ −3.6 (*c* 3.70, CHCl_3_) and [α]^25^_D_ −2.0 (*c* 1.03, CHCl_3_), respectively. Since there was only one chiral carbon in every compound, the absolute configuration of these two compounds (**2** and **3**) were also established to be *R*. Interestingly, (+)-sydonic acid had also been recently isolated from a coral-derived *Aspergillus* sp. [5], this fact suggested that fungus isolated from different marine organisms might produce different stereochemistry compounds.

(−)-5-(Hydroxymethyl)-2-(2′,6′,6′-trimethyltetrahydro-2H-pyran-2-yl)phenol (**4**) was identified as the enantiomer of deoxygenated (+)-sydonol, which was a stereoselective synthesis product possessing the positive optical rotation value, [α]^20^_D_ +40 (*c* 2.2, CHCl_3_) [18]. This compound was also isolated from the fungus *Pinus strobus* in 2011 as a racemate [14]. In this study, the optical rotation of **4** was detected as [α]^25^_D_ −2.7 (*c* 1.05, CHCl_3_). As there was just one chiral carbon, the absolute stereochemistry of **4** was established to be *S*. 

The antibacterial activity of compounds **1**–**5** was evaluated by the microplate assay [19] againsteight bacterial strains, e.g., six pathogenic bacteria *Staphylococcus albus*, *Bacillus subtilis*, *Bacillus* *cereus*, *Sarcina lutea*, *Escherichia coli*, *Micrococcus tetragenus*, and two marine bacterial strains *Vibrio* *Parahaemolyticus* and *Vibrio* *anguillarum* (Table 2). Compound **1** showed weak antibacterial activity against *E. coli* and *M. tetragenus*. Compounds **2** and **4** exhibited selective antibacterial activity. Compound **2** exhibited strong inhibitory activity on *S. albus* and *M. tetragenus* with the MIC (minimum inhibiting concentrations) values of 5.00 and 1.25 µM, respectively, and **4** on *S. albus* and *B. subtilis* with the MIC values of 5.00 and 2.50 µM, respectively. Compounds **3** and **5** showed a broad spectrum of antibacterial activity. Compound **5** inhibited five pathogenic bacteria except for *S. lutea*, and **3** exhibited significant inhibiting activity to four pathogenic bacteria and uniquely against two marine bacteria, especially to *S. lutea* with the MIC value of 2.50 µM, which was similar to ciprofloxacin used as a positive control.

**Table 2:** Tests of MIC (μM) for compounds **1**–**5** against eight bacteria.

Strains	Compounds					
	1	2	3	4	5	Ciprofloxacin
*Staphylococcus albus*	>20.0	5.00	>20.0	5.00	20.0	0.312
*Bacillus subtilis*	>20.0	>20.0	2.50	2.50	10.0	1.25
*Bacillus cereus*	>20.0	>20.0	>20.0	>20.0	10.0	0.625
*Sarcina lutea*	>20.0	>20.0	2.50	>20.0	>20.0	2.50
*Escherichia coli*	20.0	20.0	5.00	>20.0	10.0	0.625
*Micrococcus tetragenus*	10.0	1.25	20.0	>20.0	10.0	0.312
*Vibrio Parahaemolyticus*	>20.0	>20.0	10.0	>20.0	>20.0	0.160
*Vibrio anguillarum*	>20.0	>20.0	5.00	>20.0	>20.0	0.160

The isolated compounds **1**–**5** were also evaluated against the larval settlement of the barnacle *Balanus amphitrite* according to literature procedures [20]. Compound **4** inhibited larval settlement completely at a concentration of 25.0 μg/mL. However, compound **5** was found to have an obvious toxic effect on the larvae at this concentration. The other three compounds did not display antifouling activity at the same concentration. This is the first report of antifouling activity for this class of metabolites.

Compounds **1**–**5** were found to be weakly cytotoxic (IC_50_ > 50 μg/mL) against HL-60 human promyelocytic leukemia and A-549 human lung carcinoma cell lines. The acetylcholinesterase inhibitory activity of compounds **1**–**5** was also evaluated, but none of the compounds exhibited obviously inhibitory activity.

## 3. Materials and Methods

### 3.1. General Experimental Procedures

Optical rotations were measured on a JASCO P-1020 digital polarimeter. IR spectra were recorded on a Nicolet Nexus 470 spectrophotometer. ^1^H and ^13^C NMR spectra were recorded on a JEOL Eclips-600 spectrometer at 600 MHz for ^1^H and 150 MHz for ^13^C in CDCl_3_ or DMSO-*d*_6_. Chemical shifts δ are reported in ppm, using TMS as internal standard and coupling constants (*J*) are in Hz. ESIMS and HRESIMS and APCIMS were measured on a Q-TOF Ultima Global GAA076 LC mass spectrometer. HREIMS were measured on a Thermo MAT95XP High Resolution mass spectrometer and EIMS spectra on a Thermo DSQ EI-mass spectrometer. Silica gel (Qing Dao Hai Yang Chemical Group Co.; 200–300 mesh), octadecylsilyl silica gel (Unicorn; 45–60 μm) and Sephadex LH-20 (Amersham Biosciences) were used for column chromatography (CC). Precoated silica gel plates (Yan Tai Zi Fu Chemical Group Co.; G60, F-254) were used for thin layer chromatography (TLC). Semi-preparative HPLC was performed on a Waters 1525 system using a semi-preparative C18 (Kromasil 7 μm, 10 × 250 mm) column coupled with a Waters 2996 photodiode array detector, at a flow rate of 2.0 mL/min.

### 3.2. Fungal Material

The fungal strain *Aspergillus* sp. was isolated from a piece of tissue from the inner part of the freshly collected sponge *Xestospongia testudinaria*, which was collected from the Weizhou coral reef in the South China Sea in September, 2008. The strain was deposited in the Key Laboratory of Marine Drugs, the Ministry of Education of China, School of Medicine and Pharmacy, Ocean University of China, Qingdao, PR China, with the access code ZJ-2008004. The fungal strain was cultivated in 50 L liquid medium in 125 Erlenmeyer flasks each containing 400 mL medium (10.0 g of glucose, 2.0 g of yeast extract, 2.0 g of peptone in 1 L of seawater) at 27 °C without shaking for 4 weeks.

### 3.3. Identification of Fungus

The fungus was identified according to its morphological characteristics and a molecular biological protocol by 16s RNA amplification and sequencing of the ITS region. The sequence data have been submitted to GenBank, accession number HM565949. The fungal strain was identified as *Aspergillus* sp.

### 3.4. Extraction and Isolation

The fungal cultures were filtered through cheesecloth, and the filtrate was extracted with EtOAc (3 × 50 L, 12 h each). The organic extracts were concentrated *in vacuo* to yield an oily residue (3.2 g), which was subjected to silica gel column chromatography using mixtures of petroleum ether and ethyl acetate to yield five fractions (Fr.1–Fr.5). Fr.2 was further purified by Sephadex LH-20 chromatography eluting with mixtures of CHCl_3_ and MeOH (1:1), and then using semi-preparative HPLC with a C18 (Kromasil 7 μm, 10 × 25 mm) column at a flow rate of 2.0 mL/min (7:3 MeOH/H_2_O; UV detection at 210 nm, *t*_R_ of **1**: 25–26 min, **2**: 24–25 min) to yield compounds **1** (6.7 mg) and **2** (2.7 mg). Fr.3 was further purified by Sephadex LH-20 chromatography with mixtures of petroleum ether-CHCl_3_-MeOH (2:1:1) and CHCl_3_-MeOH (1:1) to obtain compounds **3** (50.6 mg), **4** (8.0 mg) and **5** (7.5 mg).

Aspergiterpenoid A (**1**): white powder; [α]^25^_D_ −4.7 (*c* 3.21, CHCl_3_); UV (MeOH) λ_max_ 194.6, 216.9, 260.4 nm; IR (KBr) ν_max _3397, 3115, 2946, 2872, 1696, 1647, 1016, 803 cm^−1^; ^1^H and ^13^C NMR see Table 1; APCIMS *m*/*z* 235.1 [M − H]^−^; HREIMS *m/z* 236.1769 [M]^•+^ (calcd. for C_15_H_24_O_2_, 236.1771).

(−)-Sydonol (**2**): white powder; [α]^25^_D_ −3.6 (*c* 3.70, CHCl_3_); UV (MeOH) λ_max_ 198.1, 275.7 nm; IR (KBr) ν_max_ 3071, 2946, 2860, 1692, 1640, 1533, 1507, 1407, 1288, 1215, 764 cm^−1^; HREIMS *m*/*z* 252.1719 [M]^•+^ (calcd. for C_15_H_24_O_3_, 252.1720).

(−)-Sydonic acid (**3**): white powder; [α]^25^_D_ −2.0 (*c* 1.03, CHCl_3_); UV (MeOH) λ_max_ 206.3, 253.3, 304.2 nm; IR (KBr) ν_max_ 3400, 2955, 1670, 1560, 1417, 1325 cm^−1^; HREIMS *m*/*z* 266.1512 [M]^•+^ (calcd. for C_15_H_22_O_4_, 266.1513).

(−)-5-(Hydroxymethyl)-2-(2′,6′,6′-trimethyltetrahydro-2H-pyran-2-yl)phenol (**4**): white powder; [α]^25^_D_ −2.7 (*c* 1.05, CHCl_3_); UV (MeOH) λ_max_ 198.1, 280.5 nm; IR (KBr) ν_max_ 3272, 3115, 3039, 2949, 2872, 1866, 1696, 1619, 1288 cm^−1^; HRESIMS *m*/*z* 249.1497 [M − H]^−^ (calcd. for C_15_H_21_O_3_, 249.1491). 

(*Z*)-5-(Hydroxymethyl)-2-(6′-methylhept-2′-en-2′-yl)phenol (**5**): white powder; UV (MeOH) λ_max_ 198.1, 236.4, 280.5 nm; IR (KBr) ν_max_ 3272, 3115, 3039, 3005, 1866, 1745, 1647, 1288 cm^−1^; HRESIMS *m*/*z* 233.1540 [M − H]^−^ (calcd. for C_15_H_2__1_O_2_, 233.1542).

## 4. Conclusions

In our continuing discovery for biological secondary metabolites from marine sponge-derived fungi in the South China Sea, this study provided a series of bisabolane-type sesquiterpenoids. The chiral compounds (**1**–**4**) possess opposite optical rotation values compared with their related analogues, which were isolated from the gorgonian-derived fungi by our group. More importantly, some of them showed selective antibacterial activity and significant antifouling activity. Compound **3** exhibited significant inhibiting activity to four pathogenic bacteria and uniquely against two marine bacteria. Further studies on the structure-activity relationship and structural modification are in progress.

## Supplementary Files

Supplementary File 1: PDF-Document (PDF, 439 KB)
