# Supplementary material for: Antibacterial Bisabolane-Type Sesquiterpenoids from the Sponge-Derived Fungus Aspergillus sp
Source: Mar Drugs. 2012 Jan 19;10(1):234–41. doi: 10.3390/md10010234 (PMC3280534; doi:10.3390/md10010234)
Supplement: Supplementary File 1: — PDF-Document (PDF, 439 KB) [file marinedrugs-10-00234-s001.pdf]

Mar. Drugs **2012**, *10*, 1-x manuscripts; doi:10.3390/md100x000x

OPEN ACCESS

Marine Drugs

ISSN 1660-3397

www.mdpi.com/journal/marinedrugs

Supplementary Materials

Figure S1. ^{1}H NMR spectrum (600 MHz, $CDCl_3$) of aspergiterpenoid A (**1**)
Figure S2. ^{13}C NMR spectrum (150 MHz, $CDCl_3$) of aspergiterpenoid A (**1**)
Figure S3. ^{1}H–^{1}H COSY spectrum of aspergiterpenoid A (**1**) in $CDCl_3$
Figure S4. HMBC spectrum of aspergiterpenoid A (**1**) in $CDCl_3$
Figure S5. HMQC spectrum of aspergiterpenoid A (**1**) in $CDCl_3$
Figure S6. APCI mass spectrum of aspergiterpenoid A (**1**)
Figure S7. HREI mass spectrum of aspergiterpenoid A (**1**)
Figure S8. ^{1}H NMR spectrum (600 MHz, $CDCl_3$) of (-)-sydonol (**2**)
Figure S9. ^{13}C NMR spectrum (150 MHz, $CDCl_3$) of (-)-sydonol (**2**)
Figure S10. HREI mass spectrum of (-)-sydonol (**2**)
Figure S11. ^{1}H NMR spectrum (600 MHz, DMSO) of (-)-sydonic acid (**3**)
Figure S12. ^{13}C NMR spectrum (150 MHz, DMSO) of (-)-sydonic acid (**3**)
Figure S13. HREI mass spectrum of (-)-sydonic acid (**3**)
Figure S14. ^{1}H NMR spectrum (600 MHz, $CDCl_3$) of (-)-5-(hydroxymethyl)-2-(2',6',6'-trimethyltetrahydro-2H-pyran-2-yl)phenol (**4**)
Figure S15. ^{13}C NMR spectrum (150 MHz, $CDCl_3$) of (-)-5-(hydroxymethyl)-2-(2',6',6'-trimethyltetrahydro-2H-pyran-2-yl)phenol (**4**)
Figure S16. HRESI mass spectrum of (-)-5-(hydroxymethyl)-2-(2',6',6'-trimethyltetrahydro-2H-pyran-2-yl)phenol (**4**)
Figure S17. ^{1}H NMR spectrum (600 MHz, $CDCl_3$) of (Z)-5-(hydroxymethyl)-2-(6'-methylhept-2'-en-2'-yl)phenol (**5**)
Figure S18. ^{13}C NMR spectrum (150 MHz, $CDCl_3$) of (Z)-5-(hydroxymethyl)-2-(6'-methylhept-2'-en-2'-yl)phenol (**5**)
Figure S19. HRESI mass spectrum of (Z)-5-(hydroxymethyl)-2-(6'-methylhept-2'-en-2'-yl)phenol (**5**)

Figure S1. ^{1}H NMR spectrum (600 MHz, $CDCl_3$) of aspergiterpenoid A (**1**)

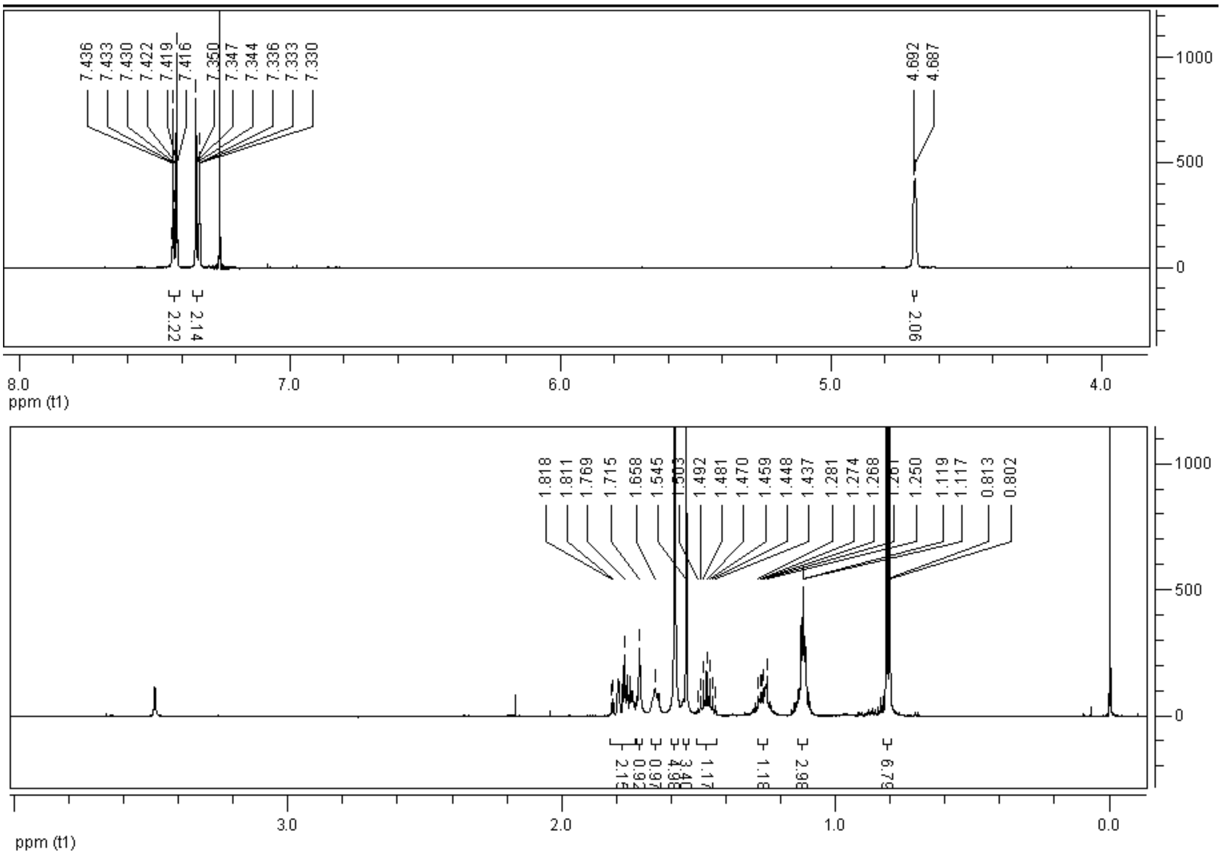

Figure S2. ^{13}C NMR spectrum (150 MHz, $CDCl_3$) of aspergiterpenoid A (**1**)

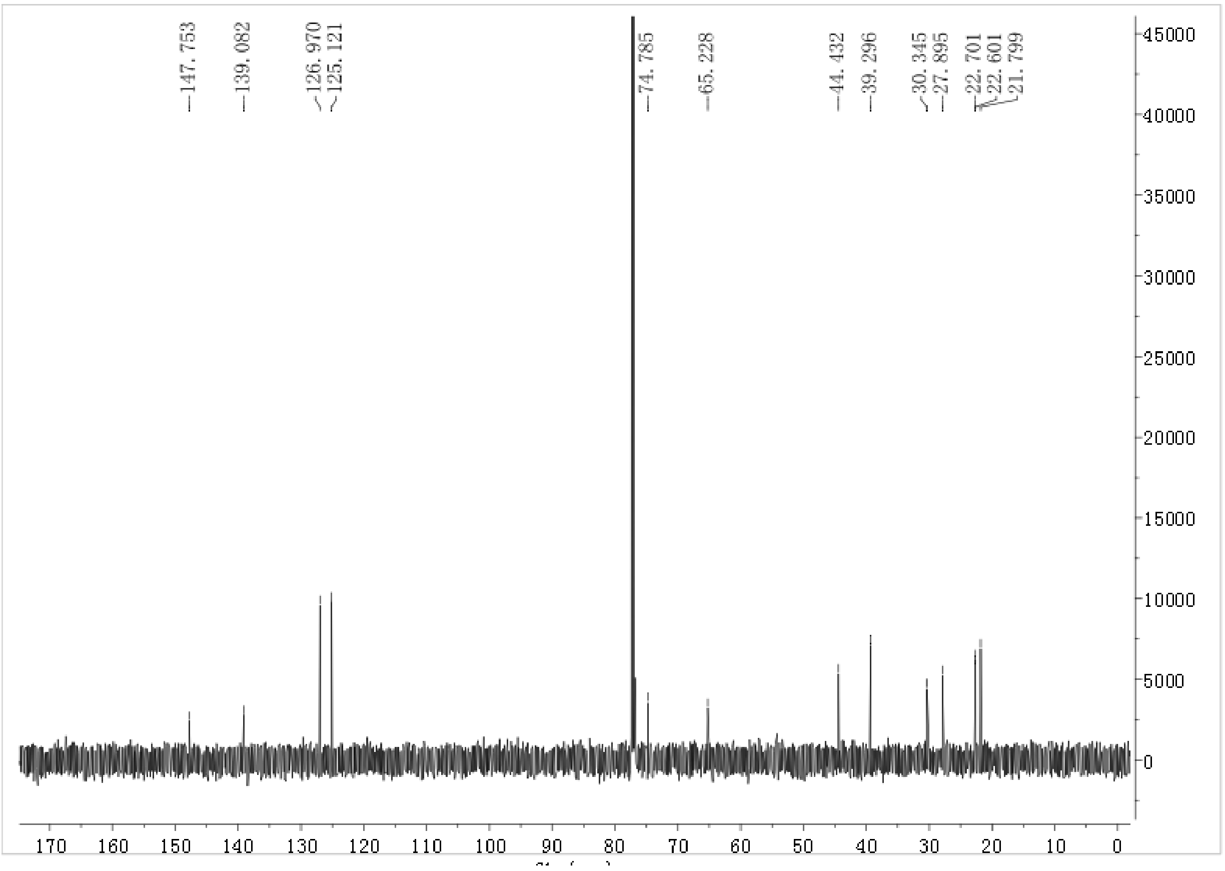

Figure S3. ^1H–^1H COSY spectrum of aspergiterpenoid A (**1**) in $CDCl_3$

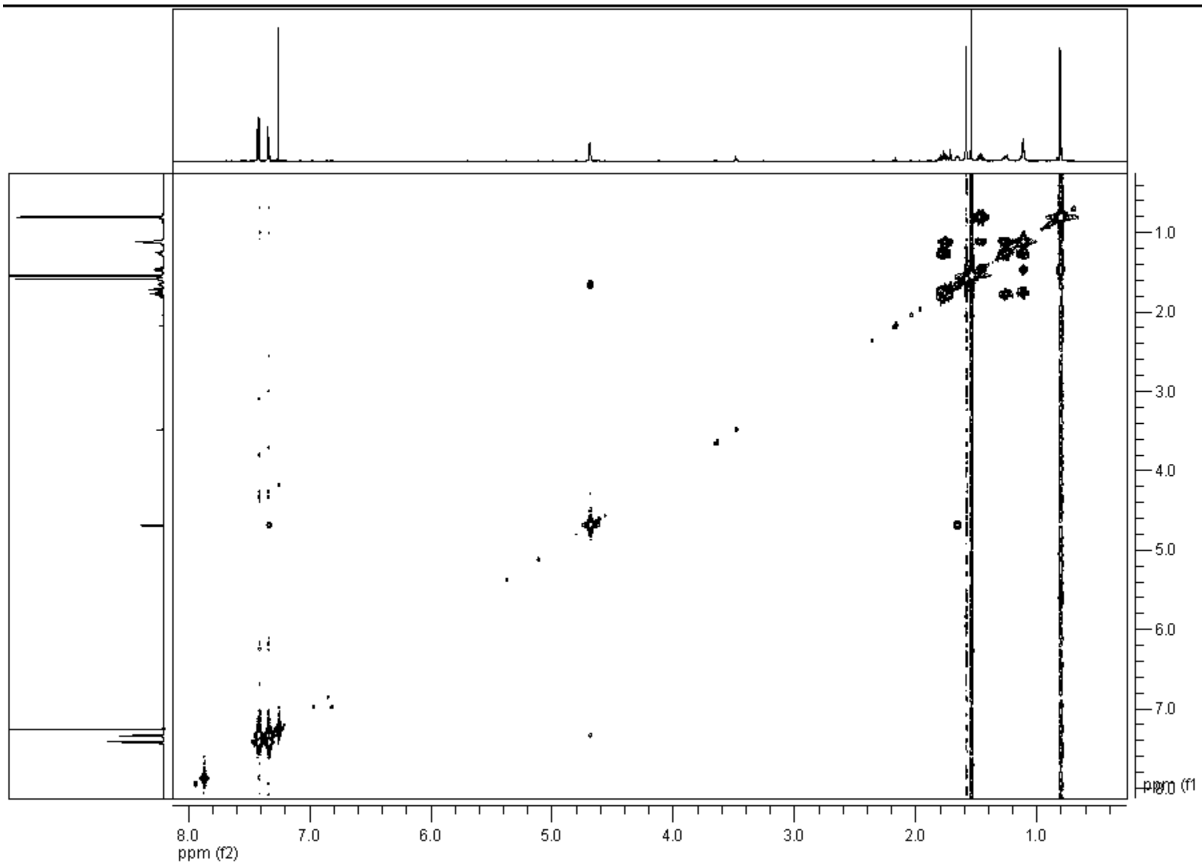

Figure S4. HMBC spectrum of aspergiterpenoid A (**1**) in $CDCl_3$

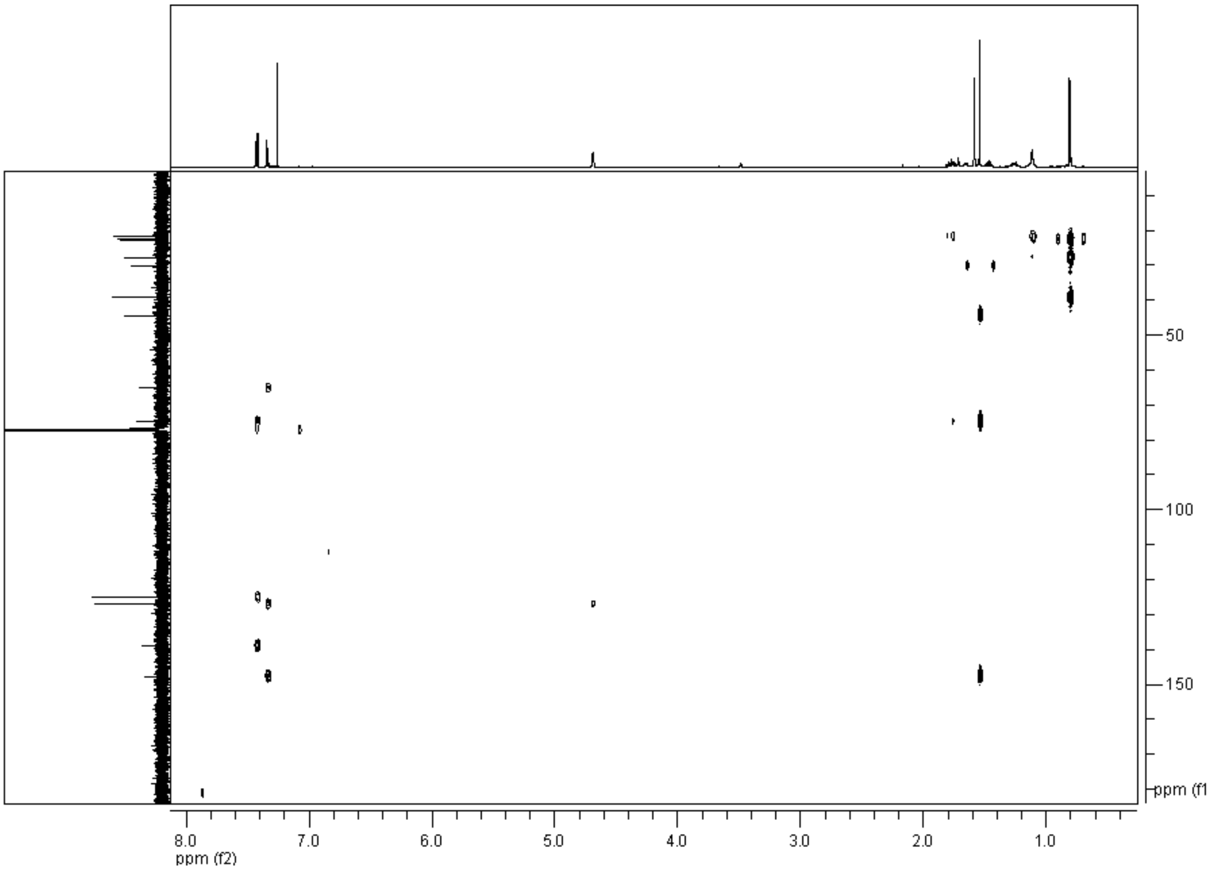

Figure S5. HMQC spectrum of aspergiterpenoid A (**1**) in $CDCl_3$

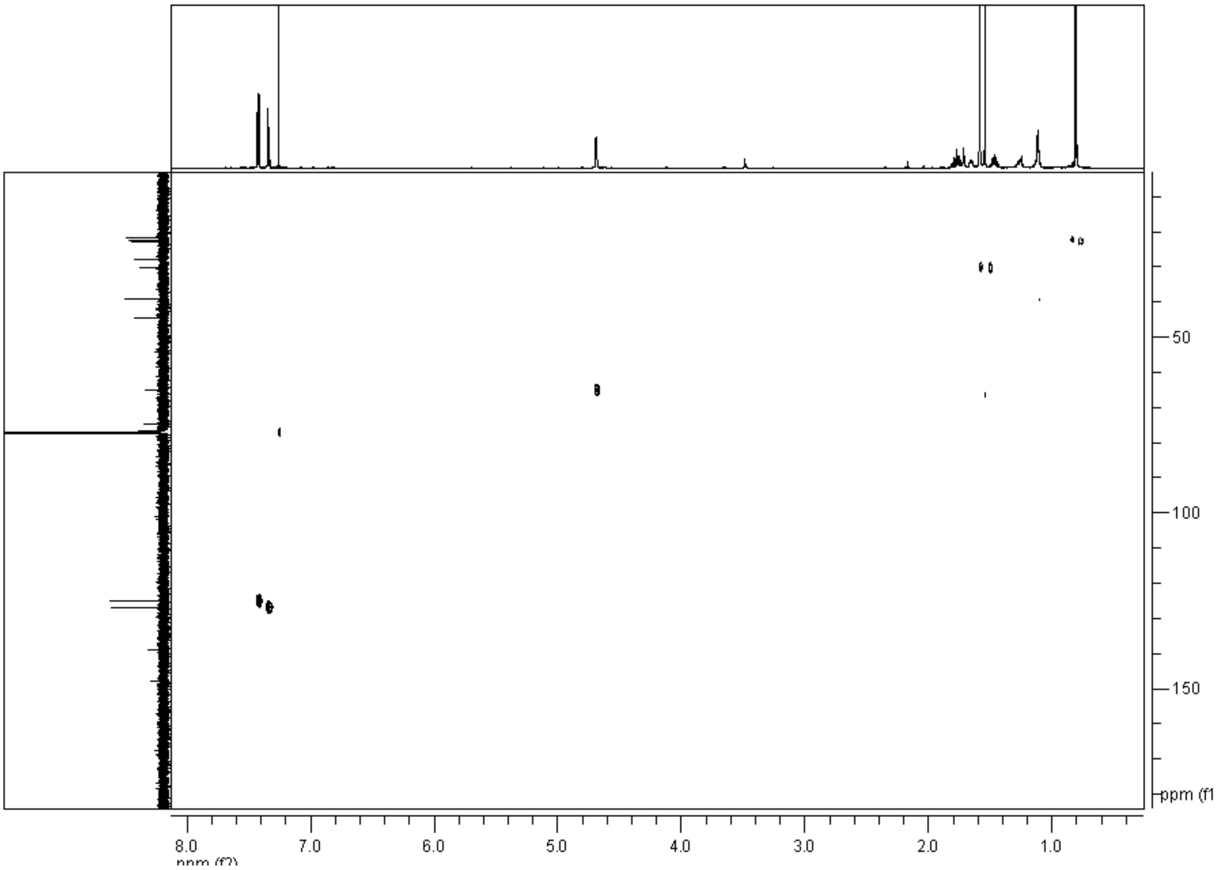

Figure S6. APCI mass spectrum of aspergiterpenoid A (**1**)

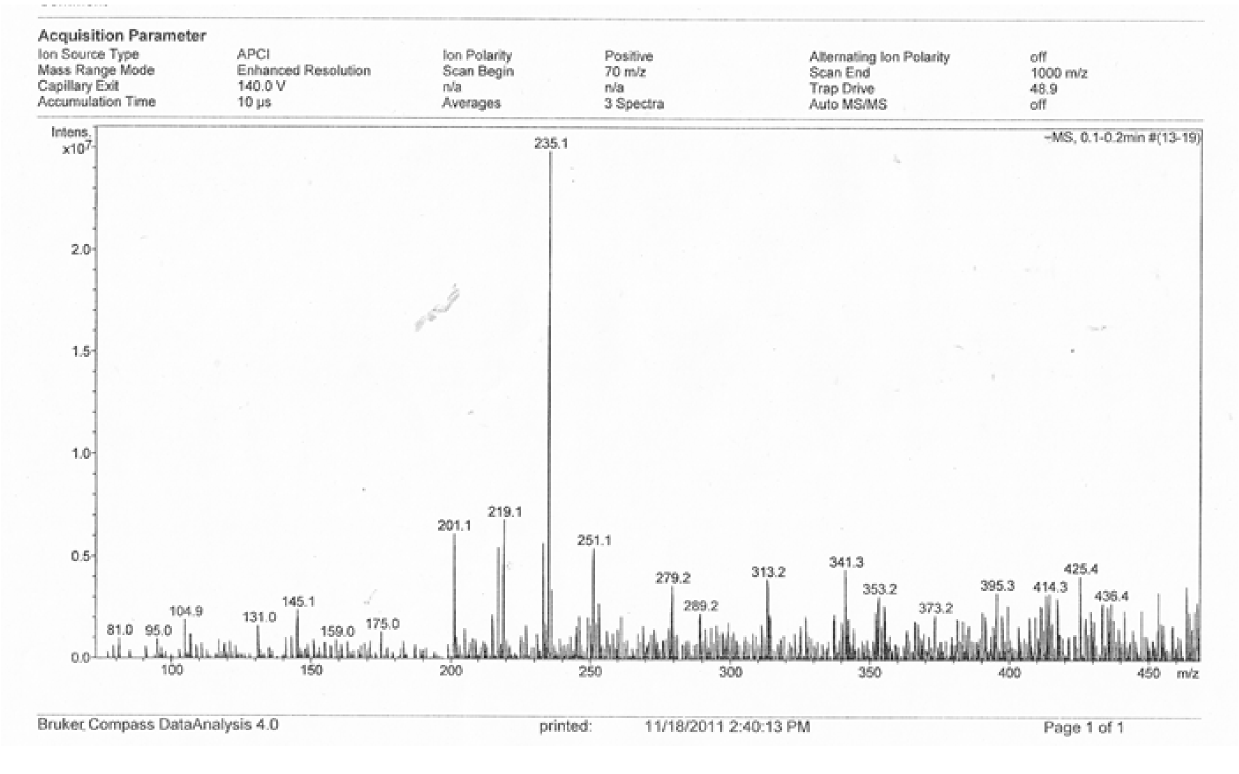

Figure S7. HREI mass spectrum of aspergiterpenoid A (**1**)

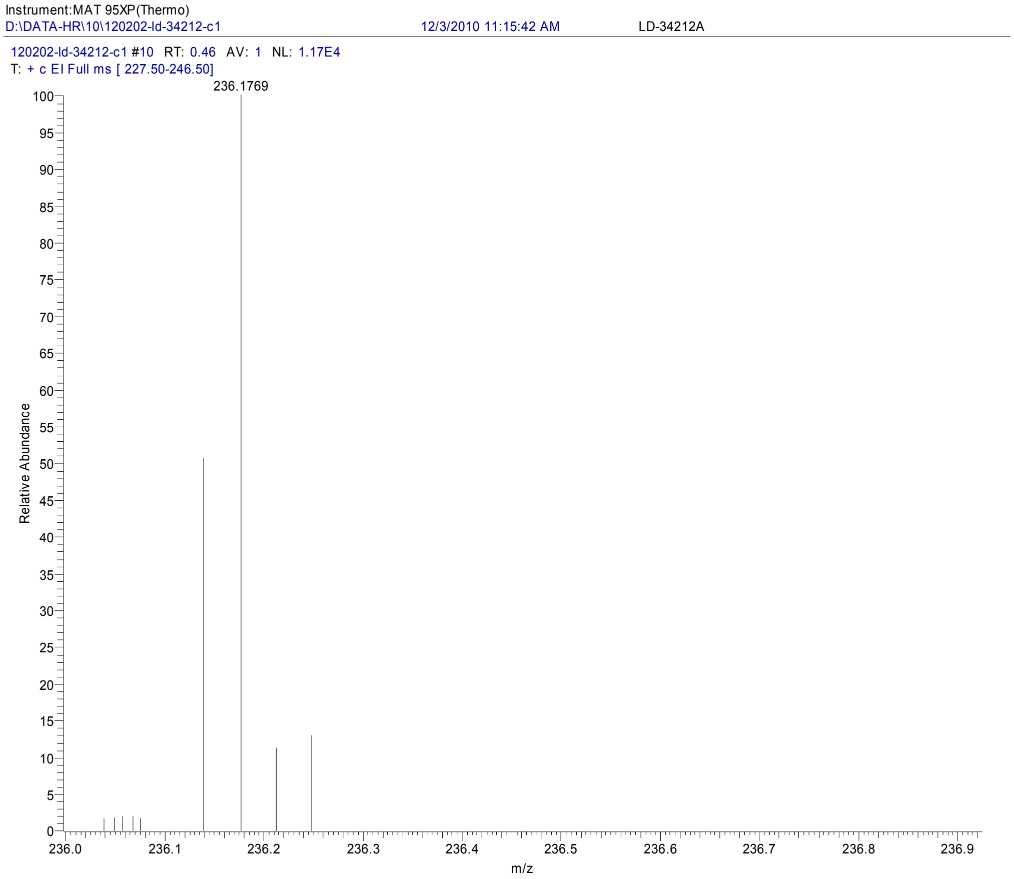

Figure S8. ^{1}H NMR spectrum (600 MHz, $CDCl_3$) of (−)-sydonol (**2**)

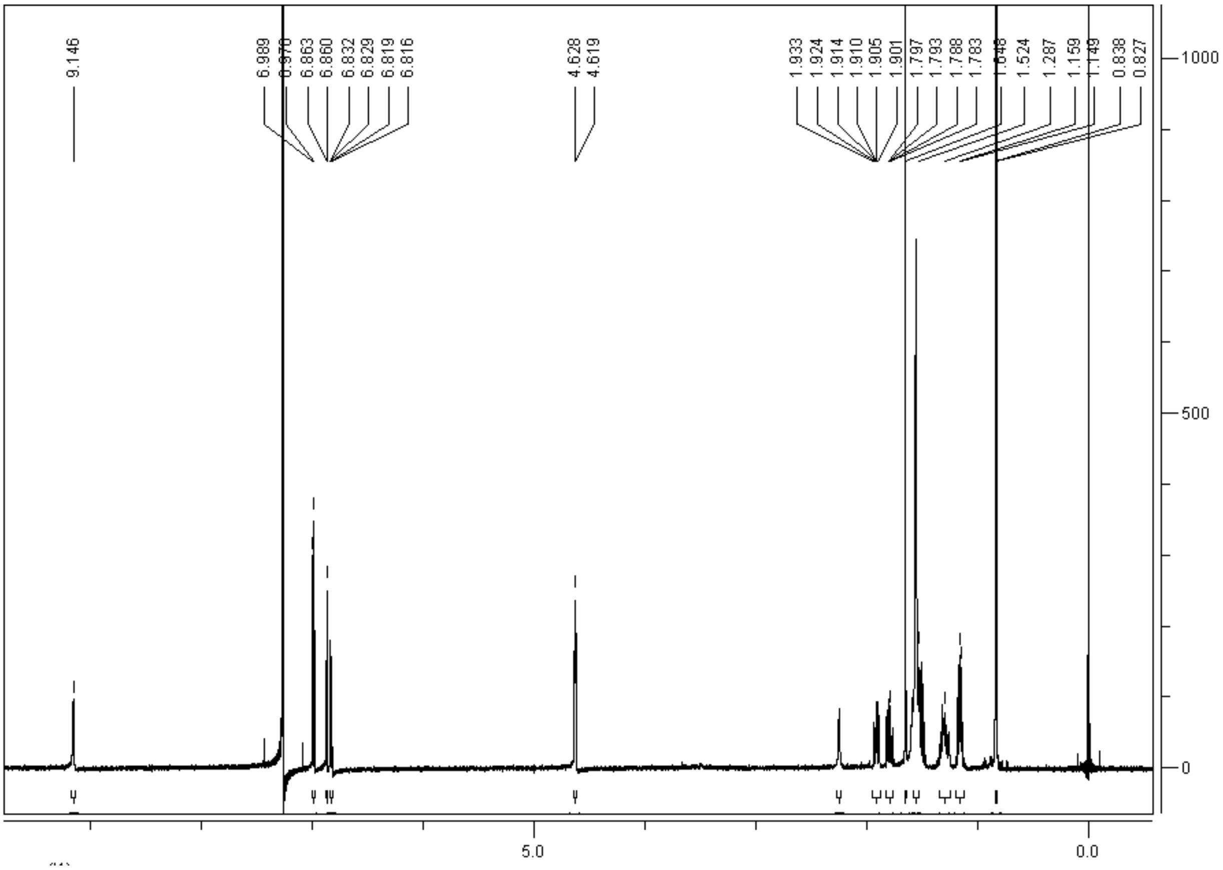

Figure S9. ^{13}C NMR spectrum (150 MHz, $CDCl_3$) of (−)-sydonol (**2**)

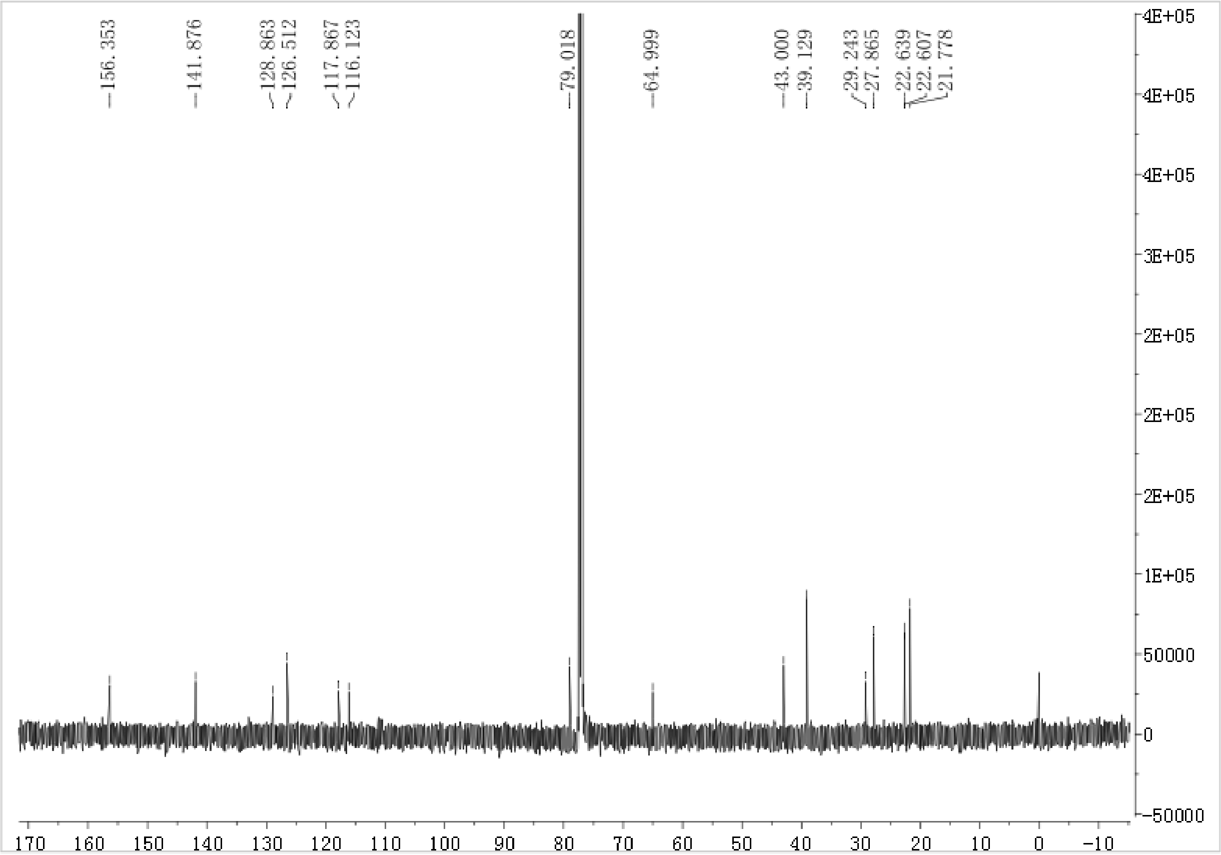

Figure S10. HREI mass spectrum of (−)-sydonol (**2**)

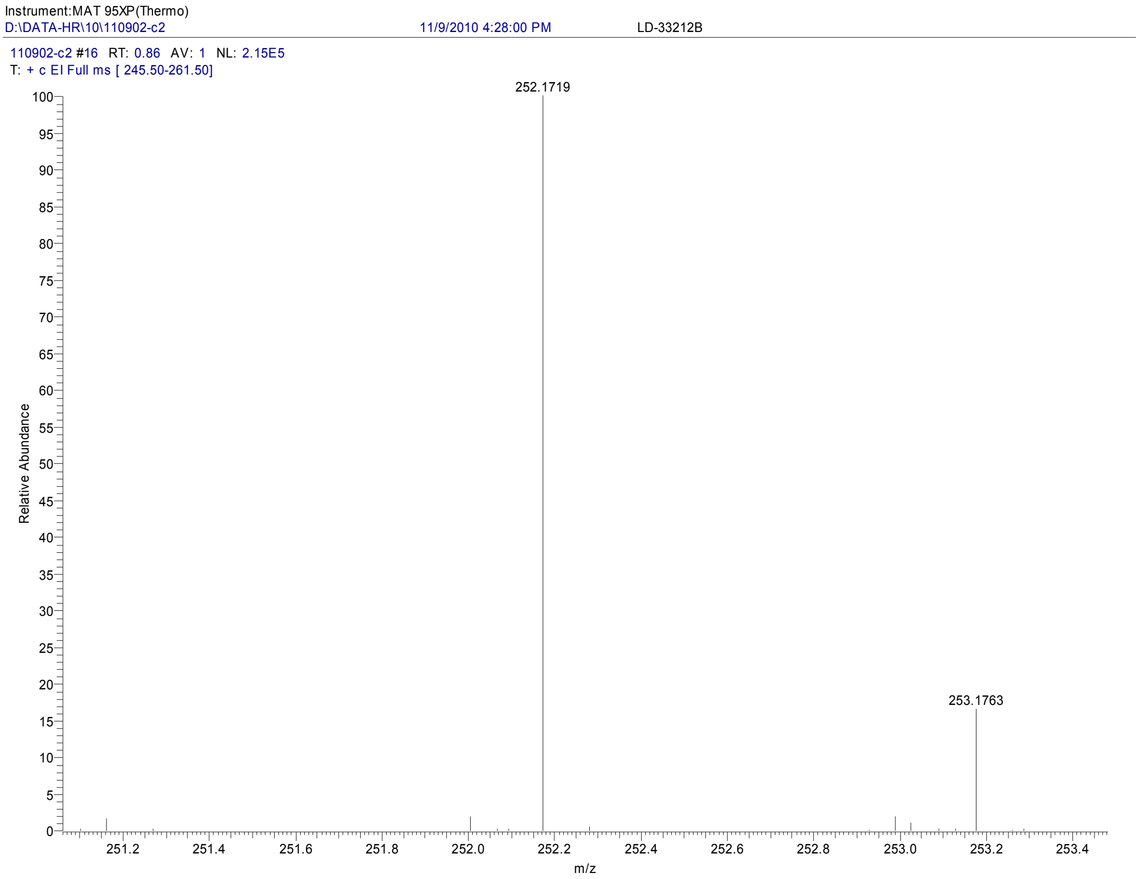

Figure S11. ^{1}H NMR spectrum (600 MHz, DMSO) of (−)-sydonic acid (**3**)

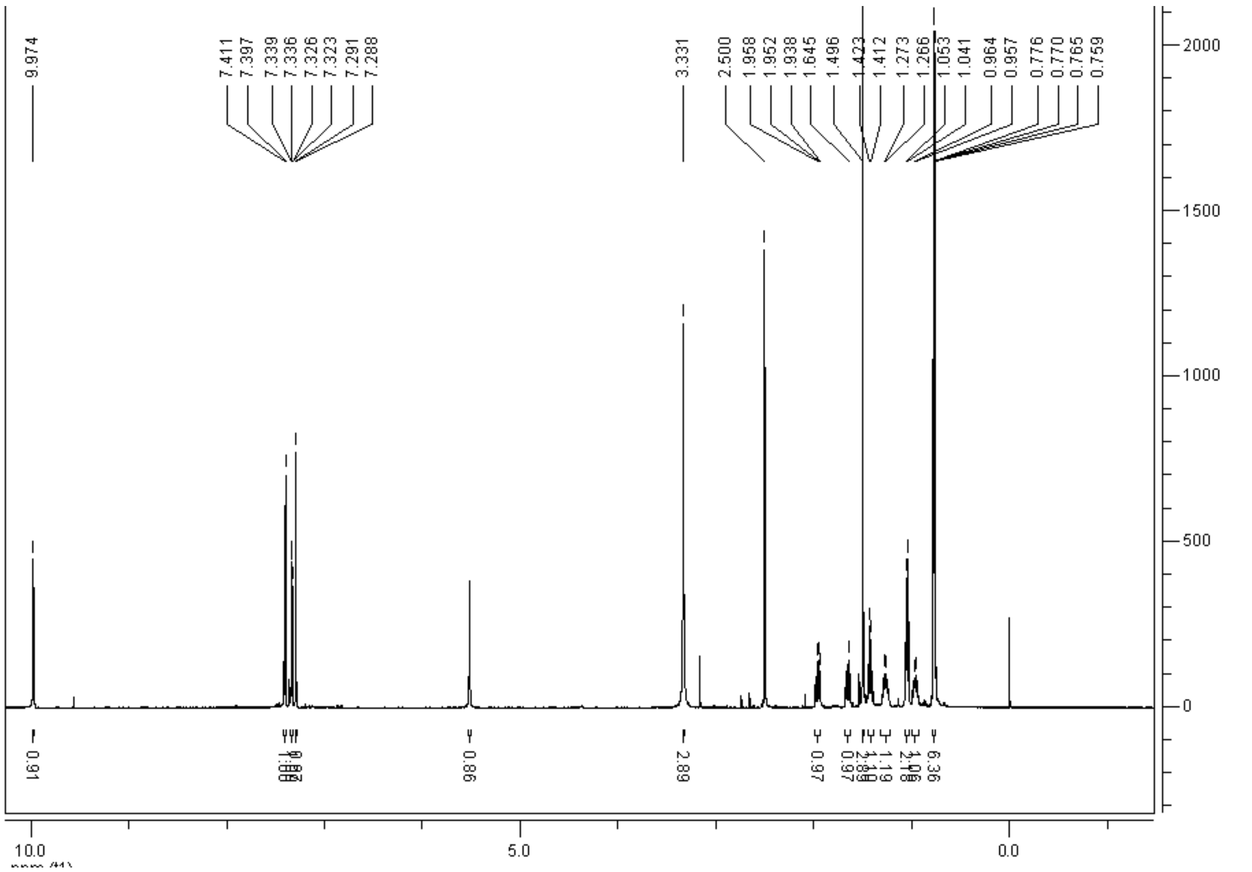

Figure S12. ^{13}C NMR spectrum (150 MHz, DMSO) of (−)-sydonic acid (**3**)

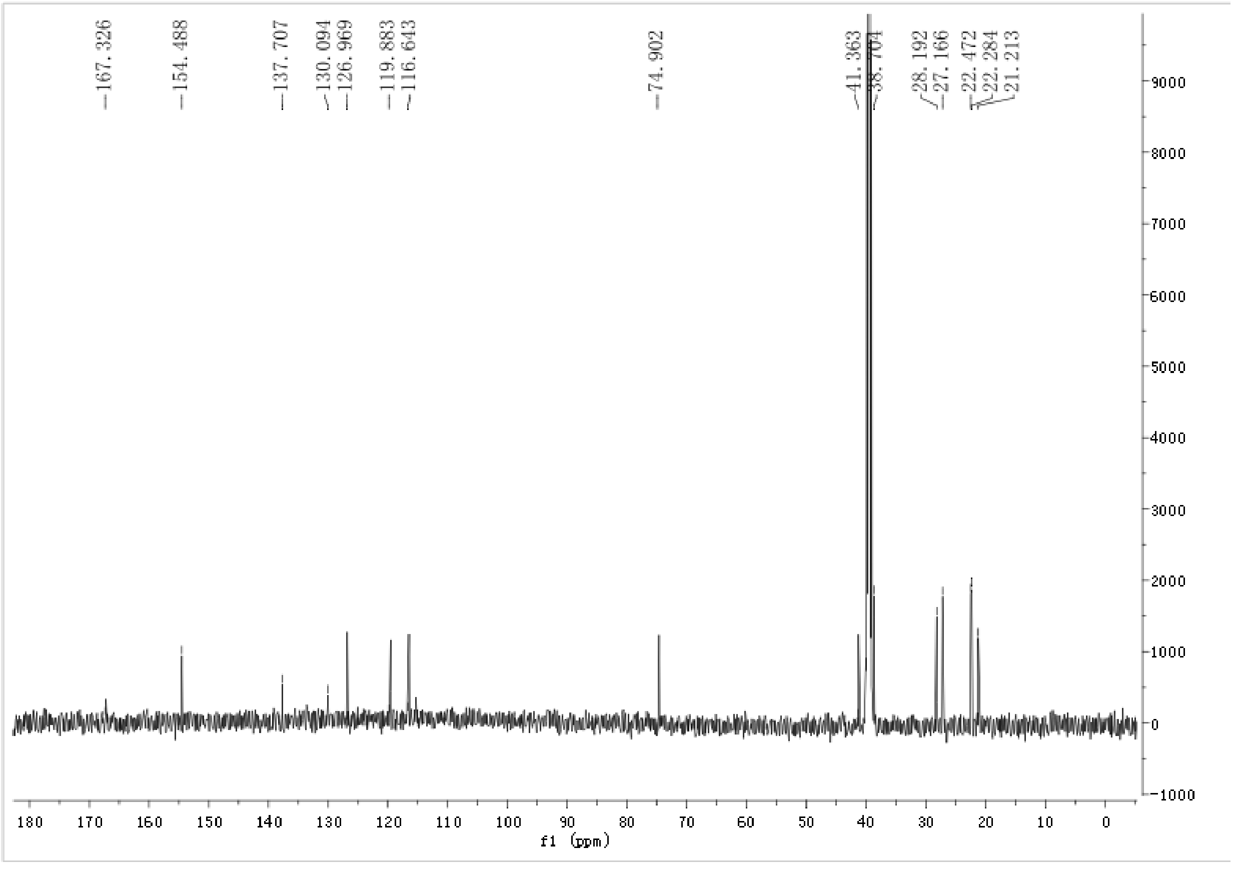

Figure S13. HREI mass spectrum of (−)-sydonic acid (**3**)

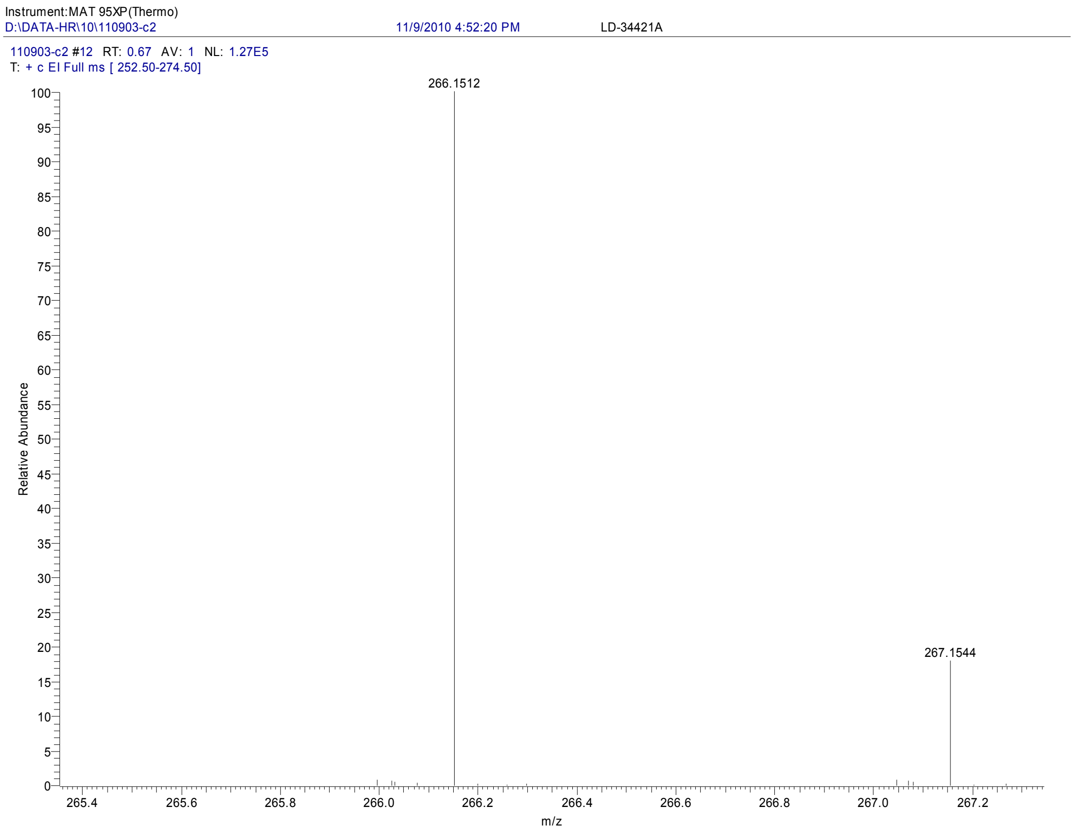

Figure S14. ^{1}H NMR spectrum (600 MHz, $CDCl_3$) of (−)-5-(hydroxymethyl)-2-(2',6',6'-trimethyltetrahydro-2H-pyran-2-yl)phenol (**4**)

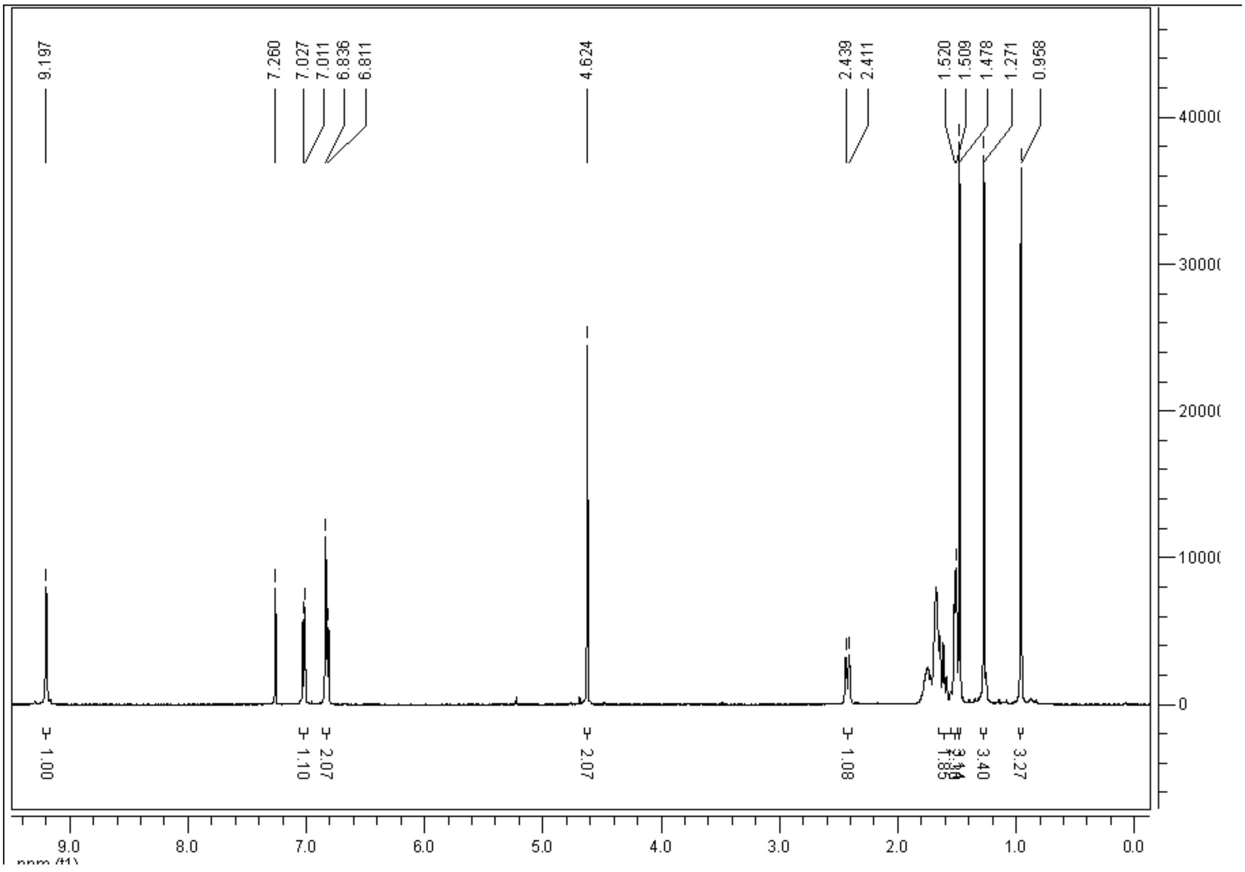

Figure S15. 13C NMR spectrum (150 MHz, $CDCl_3$) of (−)-5-(hydroxymethyl)-2-(2',6',6'-trimethyltetrahydro-2H-pyran-2-yl)phenol (**4**)

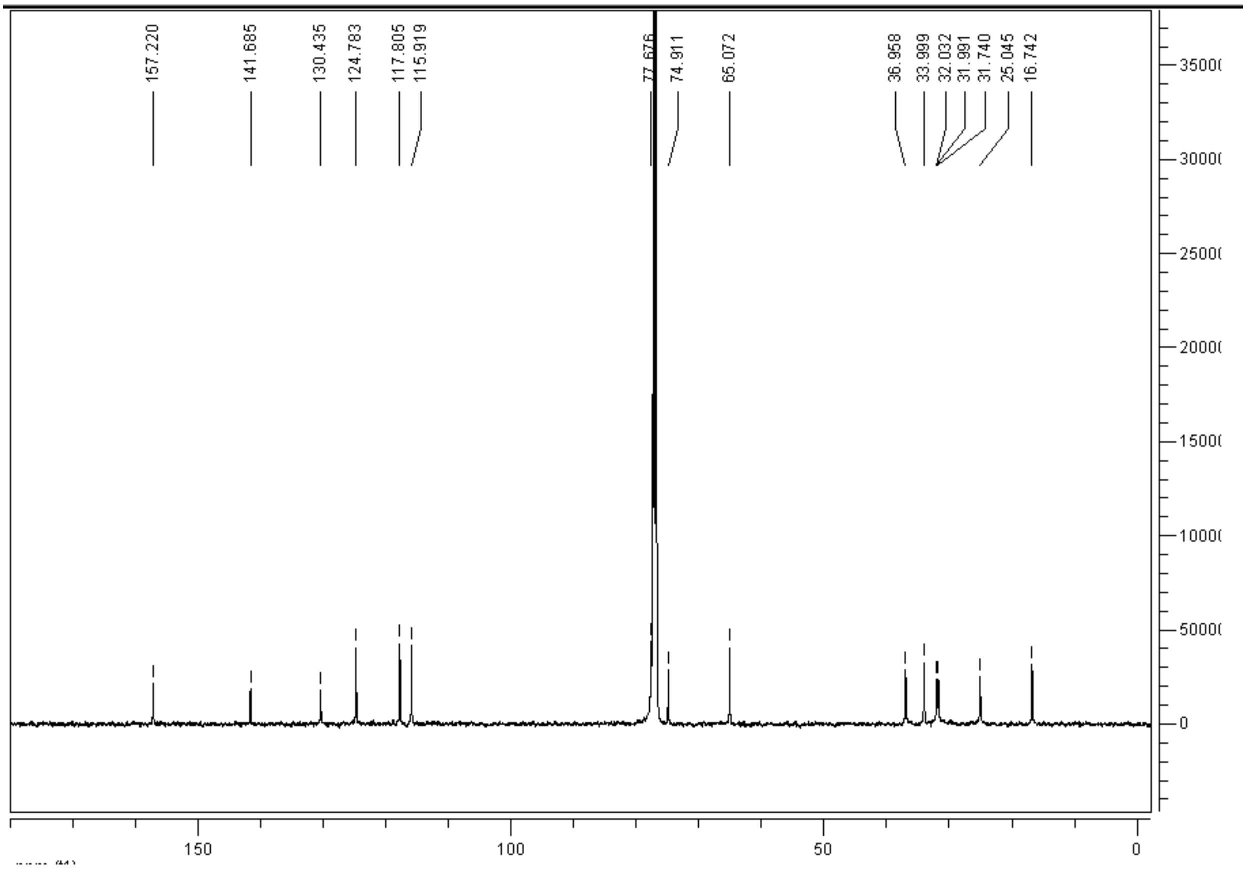

Figure S16. HRESI mass spectrum of (−)-5-(hydroxymethyl)-2-(2',6',6'-trimethyltetrahydro-2H-pyran-2-yl)phenol (4)

Elemental Composition Report Page 1

Single Mass Analysis

Tolerance = 5.0 PPM / DBE: min = -1.5, max = 50.0

Isotope cluster parameters: Separation = 1.0 Abundance = 1.0%

Monoisotopic Mass, Odd and Even Electron Ions

32 formula(e) evaluated with 1 results within limits (all results (up to 1000) for each mass)

LD-2423B

20100901-LD-2423B 62 (2.213) AM (Cen,10, 80.00, Ht,5000.0,0.00,1.00); Sm (Md, 3.00); Cm (62:69)

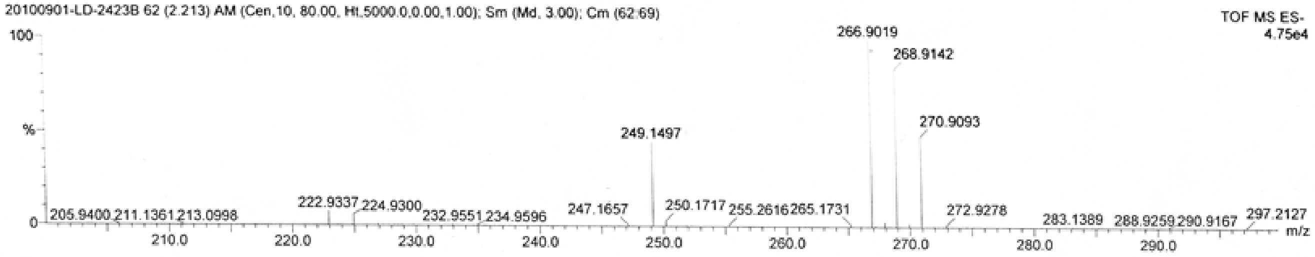

Minimum:				-1.5		
Maximum:		200.0	5.0	50.0		
Mass	Calc. Mass	mDa	PPM	DBE	Score	Formula
249.1497	249.1491	0.6	2.5	5.5	1	C15 H21 O3

Figure S17. ^{1}H NMR spectrum (600 MHz, $CDCl_3$) of (Z)-5-(hydroxymethyl)-2-(6'-methylhept-2'-en-2'-yl)phenol (5)

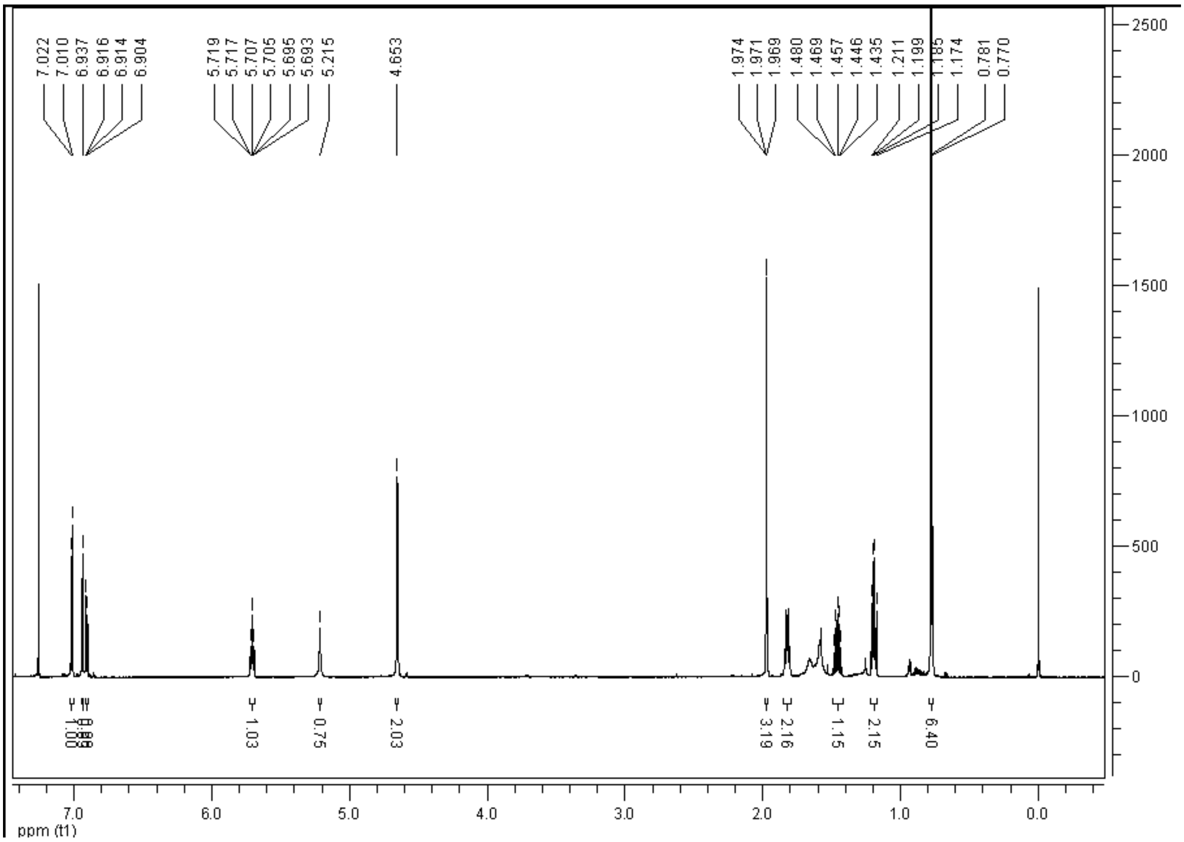

Figure S18. ^{13}C NMR spectrum (150 MHz, $CDCl_3$) of (Z)-5-(hydroxymethyl)-2-(6'-methylhept-2'-en-2'-yl)phenol (5)

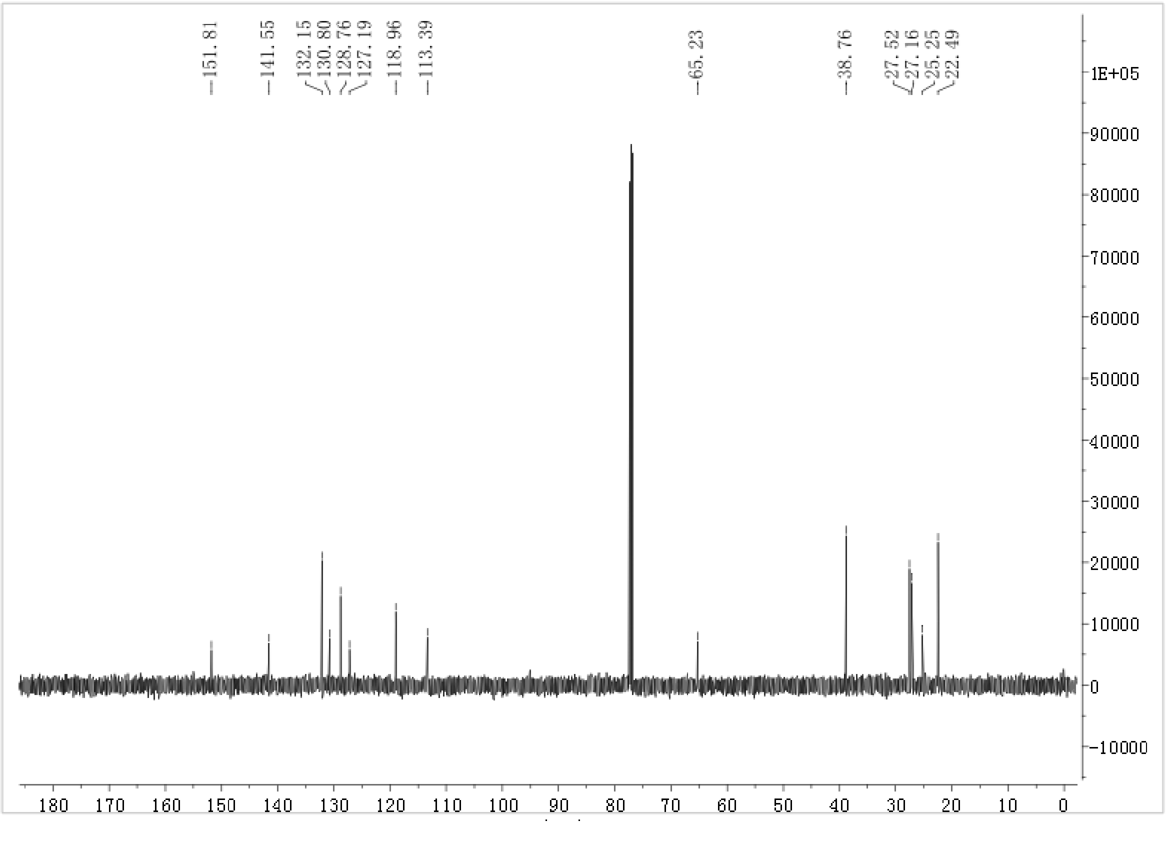

Figure S19. HRESI mass spectrum of (Z)-5-(hydroxymethyl)-2-(6'-methylhept-2'-en-2'-yl) phenol (5)

Elemental Composition Report

Single Mass Analysis

Tolerance = 5.0 PPM / DBE: min = -1.5, max = 50.0
Isotope cluster parameters: Separation = 1.0 Abundance = 1.0%

Monoisotopic Mass, Odd and Even Electron Ions
32 formula(e) evaluated with 1 results within limits (all results (up to 1000) for each mass)

LD-34231
20100901-LD-34231 71 (2.534) AM (Cen,10, 80.00, Ht,5000.0,0.00,1.00); Sm (Md, 3.00); Cm (71:75) TOF MS ES- 2.43e4

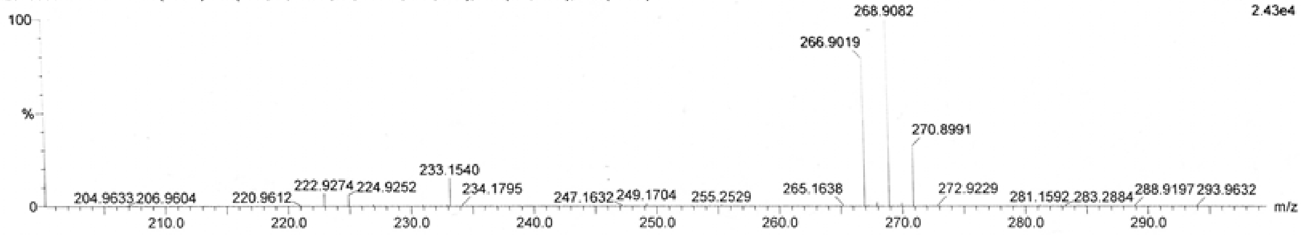

Minimum:				-1.5		
Maximum:		200.0	5.0	50.0		
Mass	Calc. Mass	mDa	PPM	DBE	Score	Formula
233.1540	233.1542	-0.2	-0.7	5.5	1	C15 H21 O2
